# Integrated Antihypertensive and Statin Treatment Protocols for Cardiovascular Disease Prevention in Low- and Middle-Income Countries

**DOI:** 10.5334/gh.1376

**Published:** 2024-12-10

**Authors:** Andrew E. Moran, Obehi Aimiosior, Reena Gupta, Anupam Pathni, Swagata Kumar Sahoo, Girma Dessie, Kufor Osi, Xiulei Zhang, Bolanle Banigbe, Renu Garg, Thomas R. Frieden

**Affiliations:** 1 Resolve to Save Lives, New York, USA; 2 Columbia University Irving Medical Center, New York, USA; 3 University of California at San Francisco, San Francisco, USA; 4 Resolve to Save Lives, New Delhi, India; 5 Resolve to Save Lives, Addis Ababa, Ethiopia; 6 Resolve to Save Lives, Abuja, Nigeria; 7 Vital Strategies, Jinan, China

**Keywords:** hypertension, dyslipidemia, statins, antihypertensive medicines, simple treatment protocol

## Abstract

In low- and middle-income countries where the majority of preventable cardiovascular disease deaths occur, less than 10% of eligible patients receive statins for primary cardiovascular disease prevention. Since 2017, the Global Hearts initiative has implemented simple World Health Organization (WHO) HEARTS hypertension and diabetes treatment protocols. In this editorial, we propose an approach of integrating statin treatment into existing HEARTS hypertension and diabetes protocols as a way of expanding statin coverage in low-and middle-income countries.

## Background: Unmet need for hypertension and dyslipidemia control in low- and middle-income countries

Globally, high blood pressure (BP) is responsible for about 10.9 million annual deaths (systolic BP > 115 mmHg; 16% of total deaths) and raised cholesterol (low density lipoprotein cholesterol > = 1.3 mmol/L) is estimated to cause 3.6 million deaths (5% of total deaths) every year (1,2). Global control of high BP and high cholesterol together can prevent a high percentage of premature cardiovascular disease (CVD) deaths and lead to longer, more productive lives for millions of people at risk. Because antihypertensive and statin treatments both reduce CVD death rate by about 25% (3,4), universal treatment of eligible patients could prevent at least one-quarter of deaths attributed to hypertension and high cholesterol, or more than three million deaths every year.

People living with hypertension and high cholesterol have higher CVD risk and benefit more from combined treatment of both conditions than people treated for only one of these conditions (5). The World Health Organization (WHO) recommends statins, and one or more of three main classes of antihypertensive medicines, as essential medicines for treatment or prevention of CVD and sets a goal for 50% of people with hypertension with BP controlled <140/90 mmHg and 50% of statin-eligible people treated with a statin. Antihypertensive medicines and statins are effective, safe, and available in low-cost generic formulations. There is substantial unmet need for these essential medicines in low- and middle-income countries (LMICs): among adults with CVD in LMICs, only 32% take antihypertensive medicines and only 16% take a statin for secondary prevention (6). For primary prevention of BP-related complications, only 31% of adults with hypertension take BP-lowering medicines and only 7% of statin-eligible patients receive statins (7). Despite the opportunity to save millions of lives through hypertension and high cholesterol control, CVD prevention medicines are not reaching people at risk, especially in LMICs.

## Integrated antihypertensive + statin treatment: A simple treatment protocol approach

Cardiometabolic diseases are inter-related physiologically and many people live with more than one chronic disease. It is therefore efficient and patient-centered for primary health care teams to screen, diagnose, and manage their patients’ chronic conditions together in a single clinic and during the same clinic visit. However, in many LMICs, primary care facilities and health information systems are designed for siloed health care—dedicated to managing only one condition (e.g., maternal/child health, HIV, or tuberculosis management)—or for managing short-term episodic illnesses and delivering one-off or infrequent services like vaccines. Chronic conditions require a chronic care model that includes chronic treatment and health information systems that monitor patient status at regular intervals over time. The challenge is to introduce integrated chronic non-communicable disease management without overwhelming already-busy primary health care teams with too many tasks and while avoiding creation of overly complex and unwieldy health information systems.

In 2016, the WHO introduced the HEARTS technical package for hypertension control, and later, a similar technical package for uncomplicated type 2 diabetes management (‘HEARTS-D’package) (8). Simple treatment protocols are the core of the HEARTS approach and scaling up treatment in LMICs where the CVD burden is highest. Simple treatment protocols are linear, dose and drug specific, and can be learned and executed by non-physician members of the health care team. These protocols improve disease control, increase fidelity to guideline recommended care, lower treatment inertia, and translate into simple, streamlined, and efficient health information systems. By 2024, over 22 million hypertension patients were managed according to simple treatment protocols in HEARTS programs in 38 LMICs (9); about one-third of these hypertension patients also have co-morbid type 2 diabetes. WHO-HEARTS significantly lowers BP and improves hypertension control compared with the usual care in LMICs (10).

**Figure 1:**
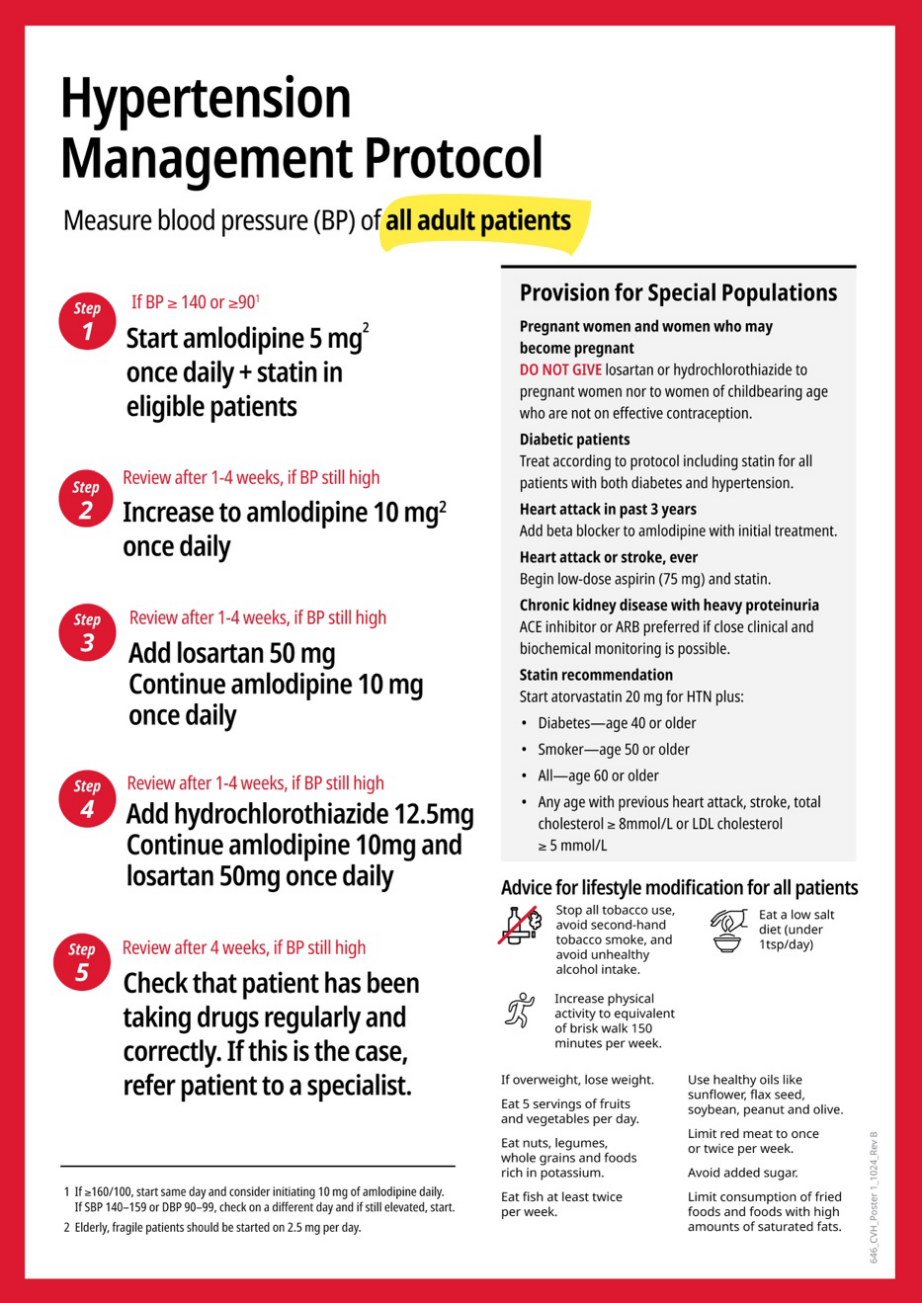
Prototype HEARTS hypertension + statin treatment protocol.

Integrating statin treatment into existing hypertension treatment protocols will provide eligible hypertension patients with both antihypertensive and statin medicines: the two treatments with greatest cardiovascular prevention benefit. An integrated protocol should not abandon a simple, linear design that minimizes cognitive overload and treatment inertia. Figure 1 shows a sample protocol that integrates statin treatment at protocol Step 1, at the same time as antihypertensive medication treatment initiation, for eligible hypertension patients. The treatment protocol design remains simple, linear, and efficient and can be followed by non-physician health workers with minimal need for re-training/up-skilling.

The WHO-HEARTS ‘D’ module recommended statin for all people aged 40 or older and living with type 2 diabetes. Statins should therefore also be integrated into HEARTS diabetes treatment protocols. However, because more than one-third of HEARTS hypertension patients also have diabetes, these patients will already receive statin as part of their hypertension treatment package.

Selected hypertension patients are recommended co-treatment with antihypertensive and statin treatment in the integrated hypertension treatment protocol. Treatment indications approximate the same CVD risk profile groups that most countries’ WHO CVD risk charts would recommend statin treatment at a threshold of ≥10% ten-year CVD risk. Indicated for at least moderate intensity statin treatment are adults aged 18 years or older with a hypertension diagnosis and the following additional characteristics:

- Diabetes—age 40 or older
- Smoker—age 50 or older
- All—age 60 or older
- Past heart attack, stroke, total cholesterol ≥ 8 mmol/L, or LDL-C ≥ 5 mmol/L—any age

Program implementers must consider primary care facility readiness requirements for implementing integrated hypertension + statin treatment protocols. Before adding statin treatment recommendations to existing hypertension treatment protocols, program implementers must ensure that health workers are trained to follow the new integrated protocol, and there is an adequate and sustained supply of statin medications. When high intensity statins are available, health care providers should prescribe them for secondary prevention of CVD or severe hypercholesterolemia. Many LMIC primary care facilities will lack lipid laboratory testing capacity; providers managing hypertension patients in these facilities can rely on non-laboratory statin treatment indications listed. Clinics with digital information systems should consider incorporating CVD risk assessment into digital, automated statin treatment decision support tools based on WHO CVD risk assessment charts. Of note, once the first treatment initiation visit is completed, health workers must re-assess hypertension patients’ statin eligibility at least once yearly at follow up visits—statin can be initiated at later follow up visits if patient factors change their eligibility (older age, new diagnosis of CVD or diabetes, high cholesterol measurement added, etc).

## Conclusion

Global delivery of two treatments to eligible patients—antihypertensive medicines to lower high blood pressure and statins to treat high cholesterol—has the potential to prevent the greatest number of CVD deaths. Expansion of HEARTS hypertension and diabetes treatment protocols to include statin treatment for eligible patients will not likely disrupt current hypertension control programs, result in complementary antihypertensive and statin medicine benefits, and be a pathfinder for integrated non-communicable disease management.
